# Supplementary material for: Oxide nanolitisation-induced melt iron extraction causes viscosity jumps and enhanced explosivity in silicic magma
Source: Nat Commun. 2024 Jan 19;15:604. doi: 10.1038/s41467-024-44850-x (PMC10799068; doi:10.1038/s41467-024-44850-x)
Supplement: Supplementary file 1 — Supplementary Information [file 41467_2024_44850_MOESM1_ESM.pdf]

Supplementary Information for

Oxide nanolitisation-induced melt iron extraction causes viscosity jumps and enhanced explosivity in silicic magma

Francisco Cáceres[1,2]*, Kai-Uwe Hess[1], Michael Eitel[1], Markus Döblinger[3], Kelly N. McCartney[4], Mathieu Colombier[1], Stuart A. Gilder[1], Bettina Scheu[1], Melanie Kaliwoda[1,5], Donald B. Dingwell[1]

[1]Department of Earth and Environmental Sciences, Ludwig-Maximilians-Universität (LMU) München. Munich, Germany

[2]Facultad de Ciencias Básicas, Universidad Católica del Maule. Talca, Chile

[3]Department of Chemistry, Ludwig-Maximilians-Universität (LMU) München. Munich, Germany

[4]Department of Earth Sciences, University of Hawai'i at Manoa. Honolulu, HI, USA

[5]Mineralogical State Collection of Munich (SNSB - Natural Science Collections of Bavaria). Munich, Germany

* Corresponding author e-mail: francisco.caceres@min.uni-muenchen.de

Contents of this file:

Figure S1 – High- and low-temperature viscosity data.

Figure S2 – Normalised magnetic hysteresis for the different steps.

Table S1 – Textural analyses of samples.

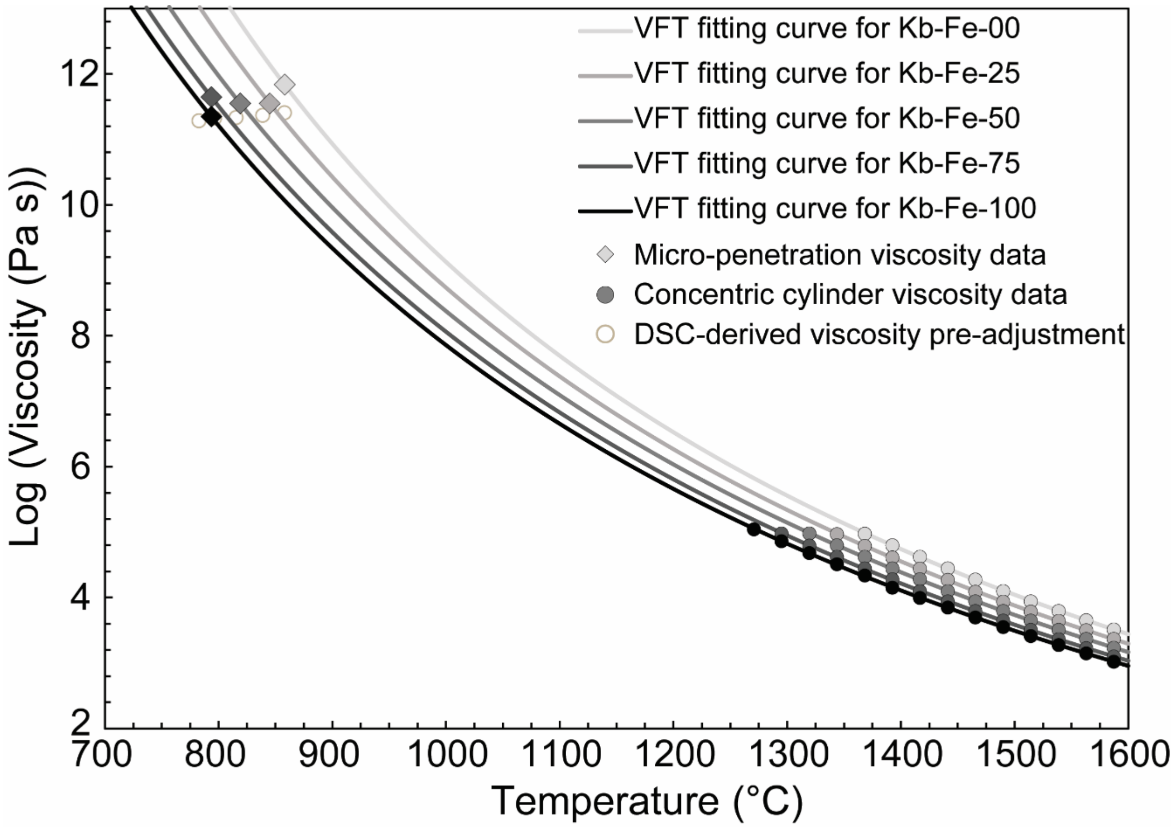

Figure S1. Viscosity data from concentric cylinder (high temperature) and micro-penetration (low temperature). Lines are VFT fittings between the high and low temperature data. Rings show the DSC-derived viscosity data used to adjust the shift factor according to the difference between the calculated viscosity and the real one represented by the VFT fittings. A systematic underestimation of the viscosity of 0.29 log units was found using the data from the iron-bearing samples only, since these compositions are closer and represent a more realistic approach for the natural sample used in this study.

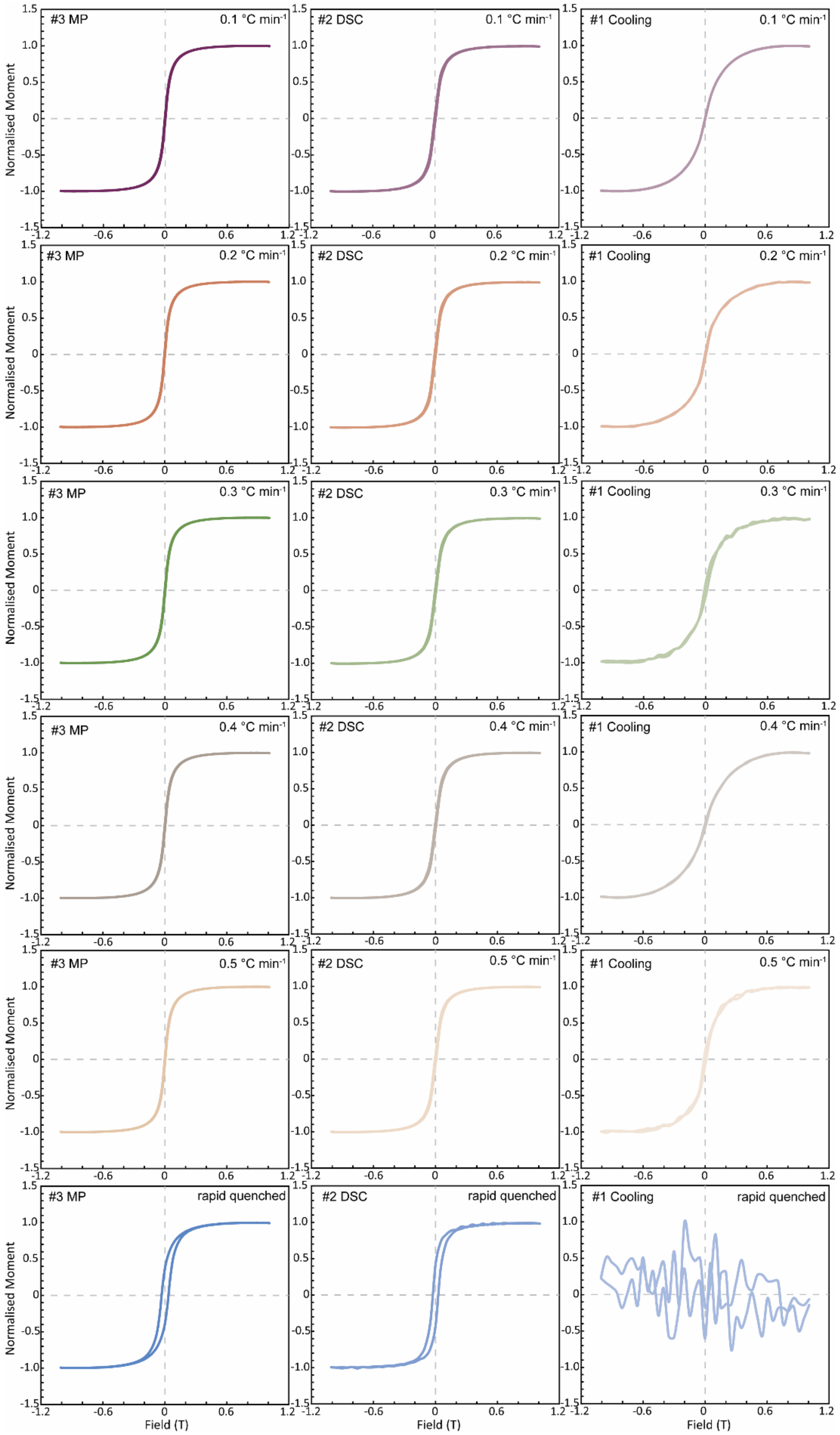

Figure S2. Normalised magnetic hysteresis analyses for each step: Cooling-controlled experiments (#1 Cooling), Differential Scanning Calorimetry (#2 DSC) and Micro-Penetration (#3 MP). Horizontally, panels show the same sample named by their initial cooling rate and after each step. Vertically, panels show different samples from the slowest to the fastest cooling rate, and the rapid-quenched sample at the bottom.

Table S1. Textural analyses. #1Cooling = after cooling experiments. #2DSC = after calorimetry analyses. #3MP = after micro-penetration analyses. CND = crystal number density of nanolites.

**Sample initial cooling rate (°C min^{-1})**	**Nanolites size range #3MP (nm)**	**Crystallinity #1Cooling**	**Crystallinity #2DSC**	**Crystallinity #3MP**	**CND #3MP (mm^{-3})**
0.1	5-13	0.11	1.05	1.11	1.93×10^{12}
0.2	-	0.03	1.07	1.12	-
0.3	-	0.02	1.08	1.13	-
0.4	-	0.01	1.06	1.12	-
0.5	5-12	0.01	1.09	1.13	1.50×10^{12}
rapid quenched	52-128	0.00	0.10	0.61	1.29×10^{8}
